# Analysis of attitudes and practices influencing adherence to seasonal malaria chemoprevention in children under 5 years of age in the Dosso Region of Niger

**DOI:** 10.1186/s12936-022-04407-z

**Published:** 2022-12-06

**Authors:** Daniel Christian Koko, Aminata Maazou, Hadiza Jackou, Charlotte Eddis

**Affiliations:** 1 U.S. PMI Impact Malaria Project, Population Services International, Niamey, Niger; 2 Programme National de Lutte Contre le Paludisme, Niamey, Niger; 3 U.S. PMI Impact Malaria Project, Population Service International, Washington, DC USA

**Keywords:** Seasonal malaria chemoprevention, Adherence, Attitudes, Practices, Distributor, Caregivers

## Abstract

**Background:**

Seasonal malaria chemoprevention (SMC) consists of the intermittent administration of a 3 day course of anti-malarial medications during the months of highest malaria risk in the Sahel region, where malaria transmission is highly seasonal. SMC is an effective intervention to reduce episodes of uncomplicated and severe malaria in children. However, morbidity cannot be lowered without adherence to medications. The objective of this study was to examine SMC medication adherence and to identify the attitudes and practices of caregivers during the 2020 SMC campaign in the Dosso region.

**Methods:**

This study was conducted based on data from independent monitoring using random cluster sampling. Adherence levels and the attitudes and practices of caregivers were evaluated using data from caregivers’ self-reports and analysed according to Bernard Vrijens’ taxonomy**.**

**Results:**

At the initiation of treatment phase, 99% of children (N = 2296) received their first administration of medication, with 90% of caregivers (N = 1436) knowing that the medications help prevent malaria. However, only 56% of caregivers (N = 1856) reported that treatment initiation took place under direct observation by the distributor. At the implementation of treatment phase, 90% of children (N = 2132) took their medication on the second day and 84% (N = 1068) took it the third day. “Forgetting,” “not having time,” and “the mother’s absence” were the main reasons caregivers gave to explain discontinuation of the 3 day course of medication.

**Conclusion:**

This simple, low-cost survey demonstrated that coverage of SMC and adherence by caregivers to completing the full 3 day medication course was high. The survey also showed that knowledge, attitudes, and practices of some caregivers regarding adherence to medications during the SMC campaign could be improved. Expanding distributors’ training, developing and providing them with tools for interpersonal communication, and strengthening supervision could lead to even higher adherence.

## Background

Malaria is a major public health challenge. With an estimated 241 million new cases and 627,000 deaths in 2020, this disease is a major cause of morbidity and mortality [1] worldwide. The World Health Organization (WHO) African Region is the most affected region with 95% and 96% of the global cases and deaths, respectively, and 77% of total malaria deaths occur in children under 5 years of age. Niger ranks among the ten most affected countries [1], reporting the 7th highest number of cases and the 8th highest number of malaria deaths in the world. The incidence of malaria in Niger is greatest in children under 5 years of age at 296 cases per 1000 inhabitants [2].

In 2012, the WHO recommended seasonal malaria chemoprevention (SMC) [3] for the control of malaria in children under 5 years of age in the Sahel region, where malaria transmission is highly seasonal. SMC consists of the intermittent administration of a 3 day course of anti-malarial medications during the months of highest malaria risk to prevent illness and death from malaria [3]. In Niger, sulfadoxine-pyrimethamine (SP) was given on day 1 of each 3 day course along with amodiaquine (AQ) given as a separate pill on day 1, day 2 and day 3. In 2020 this blister pack of four pills (1 SP and 3 AQ) was distributed door-to-door by community distributors to each child aged 3 to 59 months in a given household on that day, regardless of whether they live there all year round or are just visiting for the holidays. Four of these 3 day courses were given at 28-day intervals between July and October 2020.

SMC is recognized by the WHO as an effective intervention to reduce episodes of uncomplicated and severe malaria in children by approximately 75% [3] and there is published evidence of impact at scale under programmatic conditions [4, 5]. However, achieving this reduction in morbidity cannot be achieved without community distributors and caregivers complying with the protocol. After COVID-19 spread globally in early 2020, international and national protocols for SMC were updated to include COVID-19 adaptations to prevent the SMC campaigns from becoming super-spreader events. Unlike in previous years, when community distributors administered drugs to children themselves, they were instead asked to give the medicines to the caregivers and to instruct them to administer one SP and one AQ pill immediately, so they could be directly observed while maintaining social distance. As in previous years, the second and third AQ pills would be administered on subsequent days, preferably at the same time of day, by caregivers unobserved by a community distributor.

**Fig. 1:**
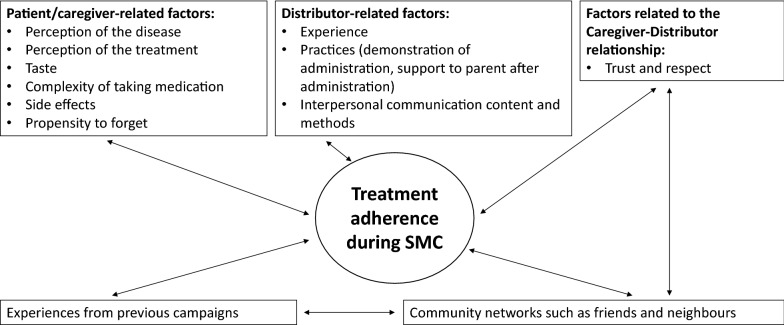
Summary diagram of factors influencing medication adherence during SMC

Adherence to a medication regimen is the process by which patients take their medications as prescribed by a health care provider. Some investigators have refined the definition of adherence to include data on taking the prescribed number of pills each day and taking the pills within a prescribed time frame [6]. Poor adherence during SMC campaigns could lead to less-than-optimal protection of the children. Adherence during SMC is determined by many factors (see Fig. 1), including patient-related factors [7, 8], distributor-related factors, and the relationship between the patient and distributor [7–10]. The latter can contribute to poor adherence when distributors do not explain how to administer the medication correctly, or the benefits and adverse drug reactions, and do not consider the living conditions of the caregivers (e.g., unavailability of clean water in the house) [11–13] during health education sessions. Community networks such as friends and neighbours that help alleviate the caregiver’s concerns and build trust in medication efficacy, and experience acquired during previous campaigns have been identified as factors related to medication adherence during SMC [10].

Despite the large volume of literature on adherence to medications, a “gold standard” has not been clearly established to measure adherence. The available methods can be categorized as direct and indirect methods. Examples of direct methods of measuring adherence are administering the medications under direct observation, measuring concentrations of a drug or its metabolite in the blood or urine, and detecting or measuring a biological marker for the drug in the blood. Direct approaches are expensive, complicated, and likely to be biased by the patient. However, for some medications, measuring these levels is a good, commonly used method to evaluate adherence. The indirect methods for measuring adherence include asking the patient about the ease with which s/he takes the prescribed medications, evaluation of clinical response, tablet counting, collecting questionnaires from patients, using electronic drug monitors, measuring physiological markers, having the patient keep a medication diary, and evaluation of the children’s adherence by asking for the help of a caregiver [14, 15]. This last technique was used during an independent monitoring survey conducted during SMC to evaluate adherence in children in the Dosso region.

**Fig. 2:**
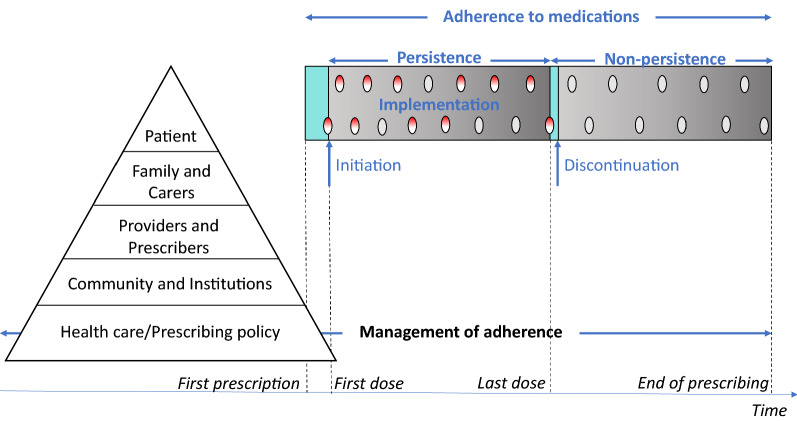
An illustration of the adherence to medications process and the management of adherence process. Source: Vrijens et al. [16]

In 2022, the National Malaria Control Programme and the U.S. President’s Malaria Initiative (PMI) through the PMI Impact Malaria project in Niger decided to take a second look at the data from the independent monitoring of 2020 SMC campaign using Bernard Vrijens’ taxonomy [16]. According to this taxonomy, adherence is based on three elements. The first is named “adherence to medications”, the process by which patients take their medications as prescribed (see Fig. 2). Adherence has three components: initiation, implementation, and discontinuation (see Fig. 2). The second element of the taxonomy is “management of adherence*”* and is the process of monitoring and supporting patients' adherence to medications by health care systems, providers, patients, and their social networks. The third element is *“*adherence-related sciences*”*. This element includes the disciplines that seek to understand the causes or consequences of differences between the prescribed and actual exposures to medications.

The hypothesis in this study was that adherence to SMC medication might be a problem in Niger. During campaign supervision missions in Niger, discrepancies were observed between the recommended/expected behaviours and those applied by the distributors (e.g., not directly observing the administration on the first day, but leaving the blister pack with caregivers with instructions to administer the pills later, if the child is absent at that moment) and caregivers (e.g., not going to a health centre to get an additional pill if the child vomits or spits out the medication on day 2 or 3). While adherence to SMC medication in Niger has been shown to be very high [17], more recent research found low drug levels in the blood of children despite caregivers reporting that they had administered the second and third AQ pills [18]. It should be noted that low drug levels can be caused by things other than adherence, including poor dosing, poor absorption, and rapid metabolism.

**Fig. 3:**
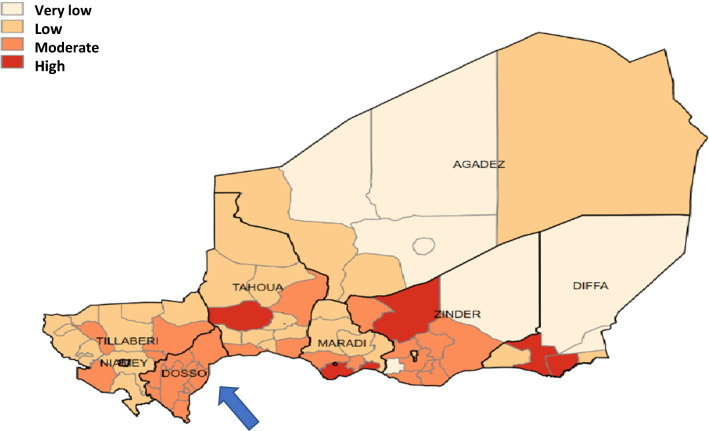
Map of the endemicity level of malaria in Niger Source: National Strategic Plan for Malaria Control 2017–2023

This study aimed to estimate adherence to anti-malarial medication in children aged 3 to 59 months during the 2020 SMC campaign in Dosso region (see Fig. 3); identify the attitudes and practices of caregivers about the administration of AQ on the second and third day; and propose corrective courses of action to improve adherence during the campaigns. To meet the study objectives, the team conducted secondary analysis of household surveys implemented during the four cycles of the campaign using a randomized cluster sampling methodology.

## Methods

This study was conducted using data collected from independent monitoring [19] of the 2020 SMC campaign in the Dosso region. Monitoring is an activity conducted independently of the implementation of the campaign with the objectives of assessing the household coverage of the intervention.

### Study area

Malaria remains a significant factor in childhood mortality in the Dosso health region. The eight health districts that make up the health region are all located in the moderate transmission area or short seasonal transmission area (less than 4 months) with an average disease incidence fluctuating between 250 and 450 cases per 1000 inhabitants [2]. With 5,144,902 cases, malaria is the first cause of consultation in health care facilities and the leading cause of mortality in Niger. The malaria fatality rate in children under 5 years of age was 0.2% in 2020 [20].

To implement the SMC campaign in the eight health districts in the region, the Dosso *Direction régionale de la santé publique* (DRSP) receives technical and financial support from the U.S. PMI Impact Malaria project.

Since 2016, Dosso has been conducting annual SMC campaigns. The implementation has increased from one health district in 2016 to all eight health districts of the region in 2019. The number of children aged 3–59 months to be reached in the intervention in 2020 was 529,380. Since 2016 when the intervention started in the Dosso region, close to 90% of the children in the intervention districts were reached with SMC. However, the incidence of malaria recorded in health centres remained high. Therefore, questions were raised about the effectiveness of SMC in the region. The caregivers’ poor adherence to the full 3 day course of medications was suggested as a possible explanation for the continued high incidence of malaria.

### Data collection

#### Study population

Caregivers of children aged 3–59 months in the Dosso region were approached and asked to complete the survey.

#### Choice of sample

The independent monitoring targeted two health districts (HD) during each cycle of the 2020 SMC campaign in the Dosso region, i.e., eight HD for the four cycles. In each HD, three health areas were randomly selected using a list of the district’s health areas. Then, a second random draw was performed from the list of localities (villages/neighbourhoods) to select three localities to be surveyed per health area. Within a locality, a random selection process was used to select twenty family compounds (note that a family compound includes more than a nuclear family e.g., it can include grandparents, multiple sons with multiple wives and their children, nephews, and younger siblings of spouses) by visiting every second family compound. In each selected family compound, the caregivers of all eligible children were interviewed systematically.

The surveys started on the second day of each cycle and were conducted for 3 days. This meant that if a child received the first dose on day 3 of the distribution period and researchers visited on that day, only questions about day 1 could be asked. Independent monitors visited the family compounds, identified eligible children, interviewed caregivers about the current cycle, and verified that the SMC cards were filled out correctly. During the four cycles, 72 localities (villages/neighborhoods) and 1440 family compounds were monitored in the eight districts of the Dosso region.

Each health district, health area, and locality were monitored only once during the campaign, i.e., the households visited after cycle 4 were not the same households as were visited after cycle 1. Nor were the households visited on day 3 the same as the households visited on day 2 of a single cycle.

The caregivers rather than the children under five were interviewed. One caregiver could respond about more than one child during a single survey. As this was programme monitoring rather than a study, no information on the age or sex of the caregiver was collected. The broad age category (3–11 months and 12–59 months) and the sex of the child was collected. A structured questionnaire was used with pre-defined answer categories. The tool was closely inspired by the survey tool used in Mali for many years. It was digitized in KoboCollect and pre-tested before going to the field.

The survey monitors were independent of the SMC campaign, in that they were recruited from outside of the health sector (from agricultural NGOs or teachers from local schools) so that the same people who were distributing or supervising the distributors would not be evaluating their own work. The household surveys were conducted for the NMCP, with funding from PMI Impact Malaria, and the reports were published as grey literature in Niger before this secondary analysis and manuscript were conceived.

### Data analysis

The project team analysed the data using the first element of Bernard Vrijens’ taxonomy [16]. This approach has the advantage of dividing the adherence process into steps and, therefore, facilitating close analysis of discrepancies that could influence the intervention. In the “adherence to medications” phase, different levels of adherence may indeed be present:

- At treatment *initiation*, for example, medications administered on the first day may not be taken under the distributor’s direct observation, or the child may refuse to drink the medication or spit out/vomit up the medication.
- *During treatment implementation*, caregivers may forget to administer the medications on subsequent days or may not administer the medications according to the dosage, or the caregiver may simply decide to stop administering the medication for some other reason (for example, after minor or major side effects occur or the child refuses to consume the other doses).

The indicators analysed were adherence to the medications on the first, second and third days of the 3 day course and the evaluation of the caregivers’ attitudes and practices.

### Description of indicators

- Percentage of target children that received the SP and AQ: number of children that received medication on day 1 of the three-day course among the total number of eligible children visited
- Percentage of the SP administration observed but not administered by the drug distributor: number of children that received medication on day 1 of the three-day course under direct observation among the total number of eligible children monitored.
- Percentage of target children that received medication on day 2 of the three-day course: number of children that received medication on the 2nd day of the three-day course among the total number of eligible children monitored
- Percentage of target children that received medication on day 3 of the three-day course: number of children that received medication on the 3rd day of the three-day course among the total number of eligible children monitored.

Note that the question on direct observation was asked in two parts: (1) If the child was treated, who administered the first dose? The surveyor was prompted to select caregiver or distributor from a drop-down box. If the answer to that first question was caregiver, a second question popped up: (2) If the dose was administered by the caregiver, was the administration supervised by the community distributor?

## Results

**Fig. 4:**
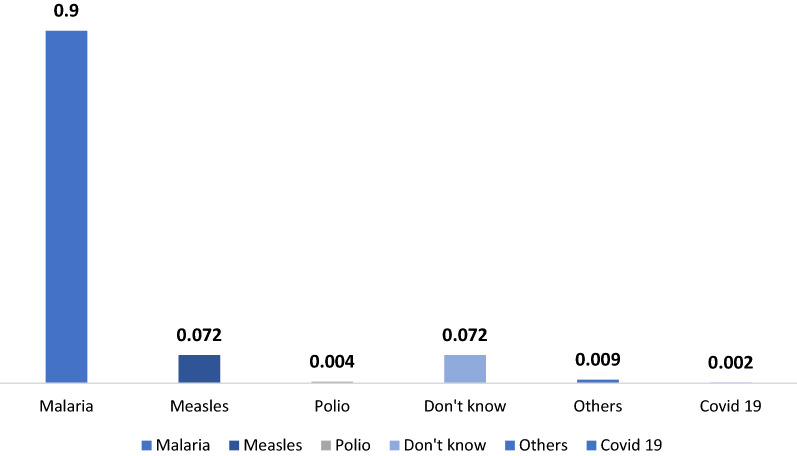
The caregivers’ level of knowledge about the disease treated during SMC (Number of caregiver respondents = 1436).

A caregiver could be a mother, father, grandmother, aunt, older sibling, or other family member or even neighbor. Regardless of their relation to the child, ninety percent of caregivers surveyed knew that the SMC medications were targeting malaria in children (see Fig. 4) (the question was: “Against which disease have your children been treated?”).

### Initiation of treatment (taking the first dose of the drug)

Evaluation of initiation of the SMC treatment by the distributors was performed in 2302 children in the Dosso region during the independent monitoring. According to the caregiver’s declaration, the proportion of children who received the first day of medication was 99.7% (n = 603) during the first cycle and remained stable at 99.0% for the other cycles. The team observed the same trends when the monitors verified that the SMC card was marked (Table 1).

**Table 1:** Proportion of children who received SP and AQ on the first day of the three-day course

	Declaration	
	n/N	%
Children who received medication on at least the first day of the three-day course in		
Cycle 1	601/603	99.7
Cycle 2	594/597	99.5
Cycle 3	560/561	99.8
Cycle 4	541/541	100.0
Cycle 1, 2, 3 and 4	2296/2302	99.7

**Fig. 5:**
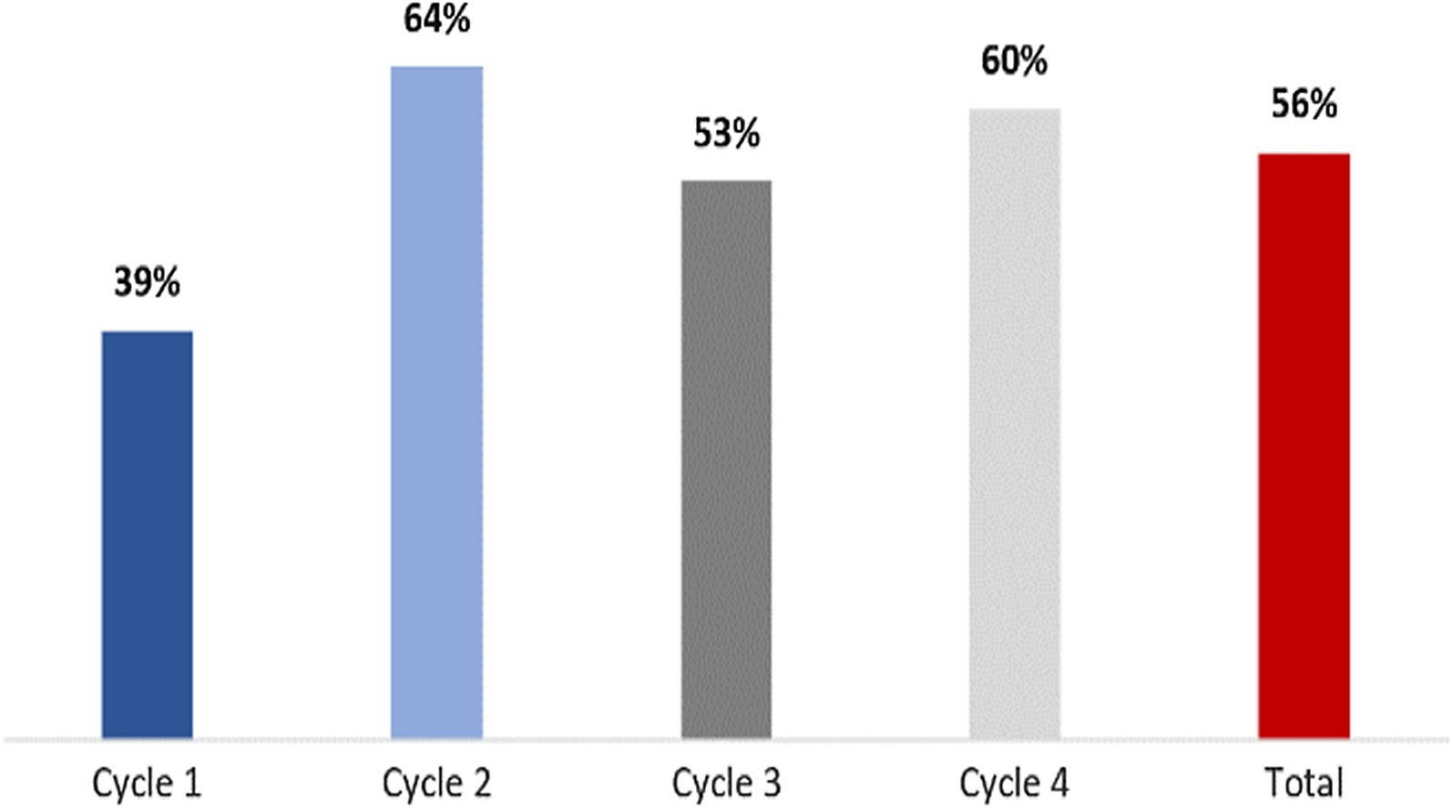
Administration of the first dose of medication under direct observation by distributors according to the caregivers’ declaration (Number of caregiver respondents = 1856)

According to 56% (n = 1035) of caregivers surveyed, treatment initiation on the first day took place under direct observation of the distributors. There is an upward trend of the observed administration of the first dose between the first cycle (39%) and subsequent cycles (Fig. 5).

### Implementation of the dosing regimen (taking medication on the second and third days of the three-day course)

A total of 3200 children were evaluated regarding adherence to the medication on the second and third days of the three-day course. According to the caregivers, 90.0% (n = 1918) of children took the AQ on the second day and 84.0% (n = 897) took the AQ on the third day of the three-day course (Table 2). Adherence to the medications on the second and third days was lower in the first cycle compared to subsequent cycles.

**Table 2:** Adherence to AQ on the second and third days as reported by the caregivers

Medication administration	Cycle 1		Cycle 2		Cycle 3		Cycle 4		Total	
	n/N	%	n/N	%	n/N	%	n/N	%	n/N	%
Second day	520/601	86.5	500/576	86.8	449/471	95.3	449/484	92.8	**1918/2132**	**90.0**
Third day	222/347	64.0	237/250	94.8	210/226	92.9	228/245	93.1	**897/1068**	**84.0**

Total values are indicated in bold

Only minor adverse drug reactions (ADR) were reported by the caregivers; 6.8% (346/5111) of children among those monitored. The most reported ADR were vomiting (27.7%), drowsiness (24.6%), diarrhoea (14.2%), and loss of appetite (11.0%). Less frequently reported ADR included the occurrence of itching (5.5%), fever (5.5%), abdominal pain (4.6%), and rash (4.6%).

### Treatment discontinuation

**Fig. 6:**
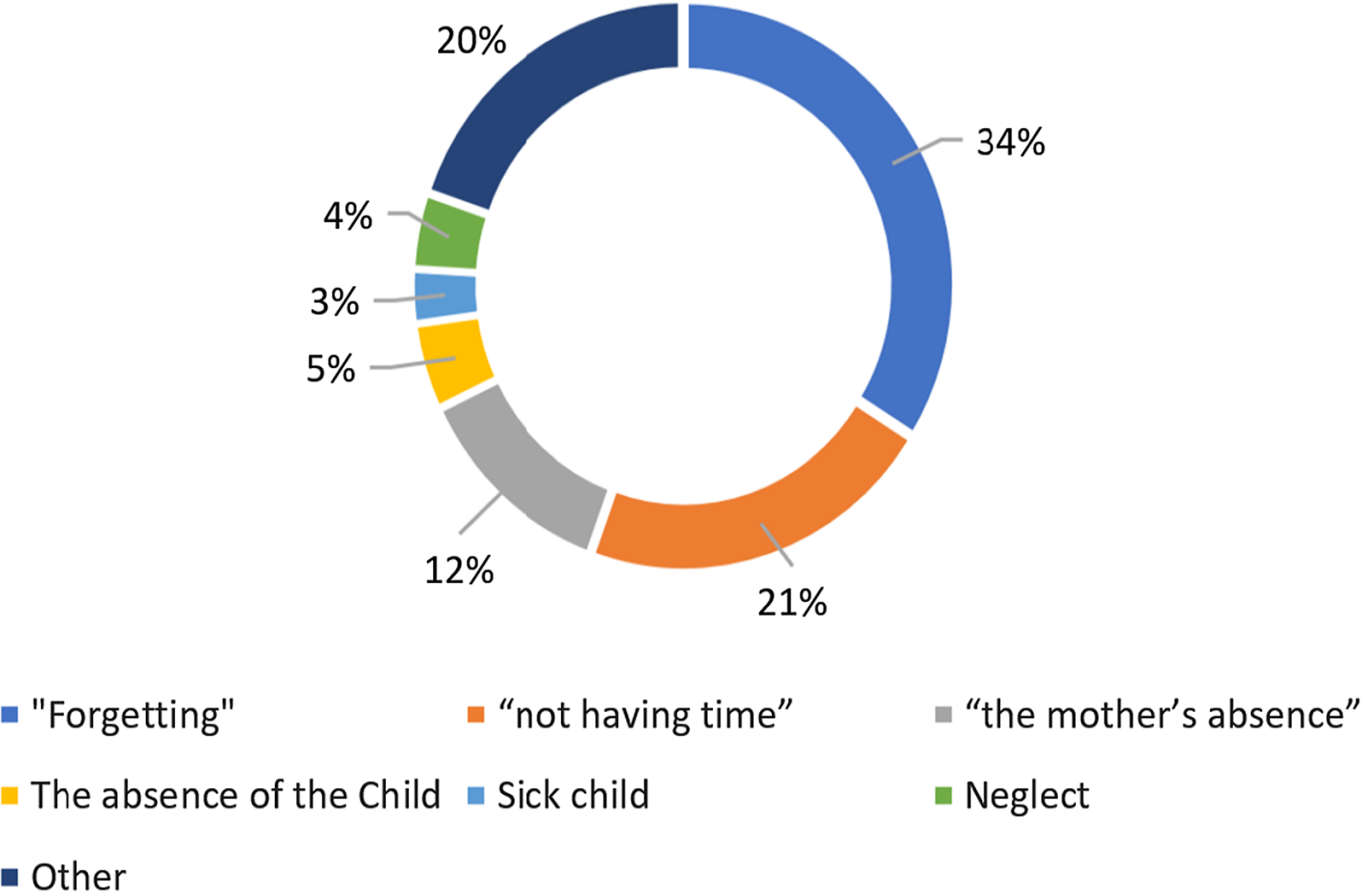
Reasons the caregivers discontinued the treatment (N = 163)

Out of a total of 1436 caregivers, only 163 (11%) interrupted their child’s treatment. The reasons were: 34% forgot, 21% did not have time and 12% were absent (see Fig. 6).

## Discussion

SMC prevents many clinical episodes of malaria in children during the high transmission season [4, 5] if the medications are administered correctly. Adherence to SMC medications is, therefore, a key factor in determining the effectiveness of the treatment. Non-adherence may also promote the spread of drug resistance [21, 22].

Analysis of the data from the health region of Dosso allowed us to identify different levels of adherence. *At treatment initiation*, 99.1% of children received the first dose of SP and AQ, which reassured us about the coverage of SMC in the target population and the acceptability of the medications by caregivers. Reported coverage levels in this study were very close to the levels found in several studies conducted in sub-Saharan Africa [9, 10, 23–27]. This high coverage could be indicative of high acceptance which could be explained by the caregivers’ knowledge of SMC as a malaria intervention, as well as by the recognized positive effect of SMC on the health of children treated [24] during previous campaigns. The good adherence to the full 3 day course seen in this study suggest that some or all these factors may have resulted from high acceptance to the intervention. However, the study by Pell et al. found that strong knowledge or positive experiences may not be needed as mothers will adhere anyway [24].

A patient’s or caregiver’s decision-making process about treatment adherence can be complicated and difficult [28, 29], even they are “aware of the risk” and motivated by the child’s well-being. Values such as the caregivers’ priorities (work or the child’s well-being), their life philosophy (treat to protect), their personal effectiveness (perceived capacity), and their history (malaria is a risk for all) are likely to contribute to the decision-making process [28]. This suggests that, although the programme is well accepted, the distributors should continue to follow the dosing guidelines and use the contact with caregivers to educate caregivers about adherence to the medications on subsequent days, potential adverse events, and what to do if they occur. Yet our results show that only 56% of caregivers report that the first dose was initiated under the distributor’s direct observation, implying that the distributors left the full blister pack in the home with instructions for someone to administer the medications later. These same practices were noted in Guinea and Senegal [30–32]. In the qualitative study by Faye et al. [31], community distributors attributed this discrepancy to a lack of time to revisit the household later in the day to see if they could arrive when the child was home, due to excessive workload.

Several studies demonstrate that caregivers have better adherence to anti-malarial treatments when the first dose is directly observed by the community distributor [33, 34] and when the caregivers have complete and accurate information [30, 32]. To overcome caregivers not having complete information, distributor training could be strengthened by role-playing typical encounters with caregivers. Then, to improve the home visits, the distributors could benefit from interpersonal communication tools (memory aids, flipcharts with images). Finally, supervision during the campaign could include observation of distributors during the encounter with caregivers with a supervision checklist assessing whether key messages were provided.

The results also showed that the level of adherence was high at the *dosing regimen implementation* level: 90% of children took their second dose of medication and 84% took their third dose, according to the caregivers’ statements. The results are consistent with the observations from the studies conducted by Diawara et al. in Senegal [26] and Tagbor et al. in Ghana [35], which evaluated adherence based on the caregivers’ statements. These levels of adherence in the Dosso region could be explained by the fact that the SMC medications were well tolerated. Only a few minor adverse drug reactions were reported by the caregivers (6.8% of children among those monitored): vomiting (27%), drowsiness (24%), diarrhoea (14%), and loss of appetite (11%). Other factors, such as the health education caregivers received during the campaign, perceived benefits of previous doses received by the children [23, 24], and a good relationship between the patient and healthcare provider, have a positive impact on adherence [36]. These details indicate the importance of providing clear and relevant information along with the medication that includes encouragement and messages that strengthen the caregivers’ good attitudes and practices.

Out of a total of 1436 caregivers, only 163 (11%) interrupted their child's treatment. “Forgetting” (34%), “not having time” (21%), and “the mother’s absence” (12%) were the main reasons caregivers gave to explain discontinuation of the SMC treatment in the Dosso region. Most people in the area studied are farmers and many of them may forget to administer the medications or may not be at home at the scheduled time to administer the medications, due to the demands of field work at that time. This observation suggests the need to develop behavioural interventions or a combination of behavioral interventions [6] to strengthen the caregivers’ adherence on subsequent days at home. These interventions could include the involvement of other family members and neighbors and other social support, such as community outreach to maximize the timely and appropriate dosing of children [37, 38].

## Limitations

As with any observational study, there is a risk of selection and observation bias. To minimize these biases, participants surveyed during the monitoring were randomly selected. The data was collected by individuals not involved in the implementation of the SMC campaign and who were unaware of the design and issue being studied.

For the interpretation of the results, certain factors must be considered. First, the adherence evaluation method of self-reporting is subject to different biases, such as memory bias and social desirability bias. To reduce memory bias, data collection took place during the campaign’s implementation, i.e., no more than one to three days after the first dose of medication. To counter social desirability bias, SMC cards and empty blister packs were also checked. Secondly, this study was conducted in the Dosso region, which mostly consists of districts located in rural areas and where the malaria burden is mixed. The results can, therefore, be generalized in regions with the same profile. However, they may be less generalizable to urban areas where caregivers have different lifestyles.

## Conclusion

This study identified the attitudes and practices that influenced adherence to anti-malarial treatment in children 3–59 months of age during the 2020 SMC campaign in the Dosso region of Niger. The study found that adherence levels were very high at treatment initiation and dropped off a bit on the second and third days of each three-day course. The main attitudes and practices influencing high levels of adherence would be the caregivers’ acceptance of the medications and good tolerability of the SMC medications, while “forgetting,” “not having time,” and “the mother’s absence” were the factors that contributed most to non-adherence. Despite the caregivers’ acceptance of SMC, distributors should continue to supervise the administration of the first dose and use this face-to-face time to strengthen the caregivers’ knowledge about adherence to the medications on subsequent days, potential adverse drug reactions, and what to do if they occur.

This study suggests that malaria programmes should carefully weigh up the potential benefit and cost based on evidence before introducing initiatives to increase full adherence such as three-day directly observed treatment. The study findings do not suggest that there is a major problem with adherence.

## Data Availability

Data has been made available on the USAID Development Data Library (DDL; https://data.usaid.gov/) as restricted access pending approval from Niger government. Data can also be requested from Population Services International by writing to https://ceddis@psi.org under a data use agreement.
